# Cathepsin X in serum from patients with colorectal cancer: relation to prognosis

**DOI:** 10.2478/v10019-012-0040-0

**Published:** 2012-07-24

**Authors:** Tjasa Vizin, Ib Jarle Christensen, Hans Jørgen Nielsen, Janko Kos

**Affiliations:** 1 Faculty of Pharmacy, University of Ljubljana, Ljubljana, Slovenia; 2 The Finsen Laboratory, Rigshospitalet, DK2200 Copenhagen, Denmark, & Biotech Research and Innovation Centre (BRIC), University of Copenhagen, Copenhagen, Denmark; 3 Department of Surgical Gastroenterology, Hvidovre Hospital, Hvidovre, Denmark; 4 Faculty of Surgery and Anaesthesiology, University of Copenhagen, Copenhagen, Denmark; 5 Department of Biotechnology, Jožef Stefan Institute, Ljubljana, Slovenia

**Keywords:** cysteine cathepsins, cathepsin X, colorectal cancer, prognosis, serum biomarker

## Abstract

**Background:**

Up-regulation of lysosomal cysteine protease cathepsin X (Cat X) is associated with disorders of the immune system and neurodegenerative diseases, while its role in the development and progression of cancer is less understood. Enhanced secretion of pro-Cat X was observed in malignant processes, and therefore, the level of total serum Cat X rather than the active enzyme may better reflect the tumour status.

**Patients and methods:**

Seventy-seven patients with colorectal cancer (CRC) were included in a retrospective study. Blood samples were collected prior to therapy. Using ELISA, the values of total Cat X were measured in serum. Groups of healthy persons (n=77), patients with adenomas (n=77) and patients with non-neoplastic findings (n=77) were included.

**Results:**

Significant differences between the group of colorectal patients and the groups of healthy persons, adenoma patients and patients with non-malignant findings could not be shown (p=0.89). Within the group of CRC, higher levels of total Cat X significantly correlated to shorter overall survival (HR=2.08, 95% CI:1.07–4.05, p=0.028).

**Conclusions:**

Total serum Cat X could be a useful prognostic indicator for determining survival of patients with CRC. Increased serum levels of total Cat X may reflect more aggressive tumour cell phenotypes and suggest the involvement of Cat X in processes involved in later stages of tumour progression.

## Introduction

Cysteine cathepsins have been reported as biomolecules that are involved in development and progression of cancer. The molecules may provide additional diagnostic and prognostic information for cancer patients.^1^–^3^ In particular, increased activity, protein concentration and secretion of cathepsins B and L have been associated with poor patient outcome in various cancer types, including colorectal cancer (CRC).^4^ Other cysteine cathepsins, including Cat X have been studied far less in cancer and their role in malignant processes is not clear. Cat X expression is restricted to various cells of the immune system, such as monocytes, macrophages and dendritic cells.^5^ It is involved in immune response regulations, such as signal transduction, growth, maturation, adhesion, cell-cell communication, proliferation and migration of immune cells and in phagocytosis.^6^–^8^ By proteolytic cleavage of C-terminal amino acids, Cat X regulates β_2_ integrin functions^9^, impairs neurotrophic activity of gamma-enolase^10^ and the role of chemokine CXCL-12 in adhesion of hematopoietic stem and progenitor cells to osteoblasts.^11^ Up-regulation of Cat X was associated also with the inflammatory processes in patients infected with *Helicobacter pylori*^12^, those with multiple trauma^13^, inflammatory related neurodegenerative disorders^10^, tuberculosis^14^ and Huntington’s disease.^15^

Several studies support the involvement of Cat X in cancer. First, the gene encoding Cat X is localized on chromosome 20 in region q13, which is frequently amplified in several types of cancer.^16^,^17^ Increased expression of the pro-Cat X and active Cat X was found in tumour and immune cells of prostate cancer^18^, gastric cancer^12^ and in the most aggressive phenotypes of human malignant melanoma.^19^ In immunohistochemical analysis of lung tumours Cat X revealed very faint staining in tumour cells, but positive staining in infiltrated immune cells.^5^ Sevenich *et al*. showed that Cat X-deficient and PymT-induced breast cancer transgenic mice had a prolonged tumour-free period for breast cancer compared to Cat X-expressing mice.^20^ Furthermore, it was demonstrated that Cat X deficiency leads to accelerated cellular senescence, a powerful tumour suppressor mechanism.^21^

Only few clinical studies related the levels of Cat X with clinico-pathological parameters in cancer patients. As shown by our group, significantly lower levels of active Cat X were found in serum of patients with inflammatory breast cancer compared to non-inflammatory breast cancer.^22^ In cyst fluid from epithelial ovarian cancer we did not find a significant correlation of the levels of active Cat X with any clinico-pathological parameter.^23^ On the other hand, increased mRNA levels of Cat X in hepatocellular carcinomas significantly correlated with advanced tumour stage.^24^

The aim of the present study was to determine the protein levels of total Cat X (pro-form and active mature form) in serum from patients with CRC in comparison to adenoma patients, patients with non-malignant findings and a group of healthy persons. Further, Cat X levels were related with clinico-pathological parameters, considering especially the correlation with overall survival of the patients.

## Materials and methods

### Patients

From October 2003 through December 2005, subjects were included in a multi-centre cross sectional study conducted at six Danish hospitals. Eligible for inclusion were subjects (aged 18+ years) undergoing endoscopy following symptoms related to CRC. Subjects previously diagnosed with CRC and subjects unable to give informed consent were excluded from the study. The Regional Ethical Committee and The Danish Data Protection Agency approved the study. Following oral and written consent according to the Helsinki II Declaration 5,165 individuals were included consecutively. Based on this study-population a case-control study was designed including 312 patients representing four diagnostic groups of subjects. The study population was selected in order to test the diagnostic potential of different promising serological markers in CRC. Primarily 77 subjects with pathologically verified colorectal adenocarcinomas (26% RC, n=20 and 74% CC, n=57) were selected at random. These patients underwent surgery and did not receive radio- or chemotherapy. For each of these, a subject with pathologically verified adenomas was randomly selected matching for age, gender and localisation of finding. Then subjects with other (non-malignant) findings were randomly selected and matched as described for the adenomas. Finally, subjects who had no findings at endoscopy and who reported no co-morbidity (healthy persons), were randomly selected and matched to the groups by age and gender. All pathological diagnoses were validated using the Danish National Registry on Pathological Examinations (www.patobank.dk). Patients who were diagnosed with cancer, but did not have the tumour removed were classified according to the clinical information available, and as a result exact tumour staging was not possible in few cases. Similarly for patients diagnosed with adenomas, the type of adenoma was not specified in all cases, either due to incompleteness of the pathological description available or due to loss of specimen during the examination. Previous cancer diagnoses were retrieved from the Danish Cancer Registry. One subject was shown to have a previous CRC and was excluded from the study together with the three matched subjects, leaving a study population of 308 subjects. Descriptive statistics for the matched subjects are given in Table 1.

### Sampling

Blood samples were collected at the same day period prior to endoscopy in all subjects, following a standard operating procedure (SOP). At sample collection subjects were non-fasting. All subjects undergoing colonoscopy had completed a bowel preparation. Blood was collected at moderate tourniquet pressure, in 10 ml serum tubes (Vacutainer^®^ Becton-Dickinson, Mountain View, CA, USA), and spun for 10 minutes at 3000 *g* and 4°C within 1 hour following collection. Samples were immediately stored at −80°C and were thawed and refrozen once prior to analysis.

### Determination of Cat X

Human total Cat X was analyzed using ELISA, as described.^5^ Briefly, microtiter plates (Nunc-IMMUNO™ MODULES, Roskilde, Denmark) were coated with goat polyclonal antibody against human Cat X (R&D SYSTEMS^®^, Minneapolis, USA), which recognizes pro-form and mature Cat X (2 μg/mL in 100 μL of 1 mM Na_2_CO_3_, 35 mM NaHCO_3_, 15 mM NaN_3_, pH 9.6). Following incubation over night at 4°C, the plates were washed three times with the washing buffer (0.15 Mm NaCl, 7.5 mM Na_2_HPO_4_ X 2H_2_O, 2.5 mM NaH_2_PO_4_ X 2H_2_O, 0.05% Tween^®^ 20 (Sigma-Aldrich Chemie GmbH, Steinheim, Germany), pH 7.2), and blocked with 2% BSA in washing buffer for 1 h. The wells were filled with Cat X standards or serum samples and incubated for 2 h at 37°C. Finally, after washing, 100 μL of horseradish peroxidase-conjugated mouse 3B10 mAb, recognizing the pro-form and mature Cat X (1:1000 dilution) was applied. After 2 h incubation at 37°C, 200 μL of TMB (3,3′,5,5′-tetramethylbenzidine (TMB) Liquid Substrate System, Sigma-Aldrich Chemie GmbH, Steinheim, Germany) in the presence of hydrogen peroxide was added. After 20 minutes the reaction was stopped with 50 μL of 2 M H_2_SO_4_. The absorbance at 450 nm was measured with a microplate reader (TECAN Saphyre 2, Männedorf, Switzerland) and corrected for the absorbance of controls. Pro-Cat X used as a standard, was prepared and characterized as described.^25^ The intra-assay and inter-assay coefficients of variance (CV) were 9.8% and 11.0%, respectively. To determine the linearity of ELISA, serum samples were serially diluted to the levels encompassing the range of the assay. Finally, the samples in a 1:2 dilution were added to the wells of microtiter plate. Detection limit of the assay was 2.0 ng/ml.

### Statistical analysis

Statistical analysis was carried out using SAS (v9.2, SAS Institute, Cary, N.C., USA). To identify a diagnostic value of total Cat X, a linear model comparing the level of total Cat X between the four groups was used. Cat X concentrations were log transformed for statistical analysis. P-values less than 5% were considered significant.

For survival analysis, the Kaplan-Meier methods, dichotomizing the values using their respective medians, and Cox regression analysis with the total Cat X values entered as continuous variables in the log scale (base 2), were used. The hazard ratio (HR) for the latter was for a two-fold difference in marker levels.

## Results and discussion

CRC is the second most frequent malignant disease in the developed countries.^26^,^27^ Besides carcinoembryonic antigen (CEA), several other serum tumour markers have been suggested for early detection of the disease and for prediction of prognosis^28^–^31^, however, none of them has reached the regular clinical use yet. The levels of serum Cat X, evaluated in this pilot study, revealed no significant difference between CRC patients and control groups, however, higher levels in CRC patients correlated with shorter overall survival and may provide prognostic information.

The levels of total Cat X in the CRC group, adenoma patients, patients with non-malignant findings and the group of healthy persons are shown in Table 2. The results show that there is no significant statistical difference in Cat X levels between the groups (p=0.89).

**FIGURE 1:**
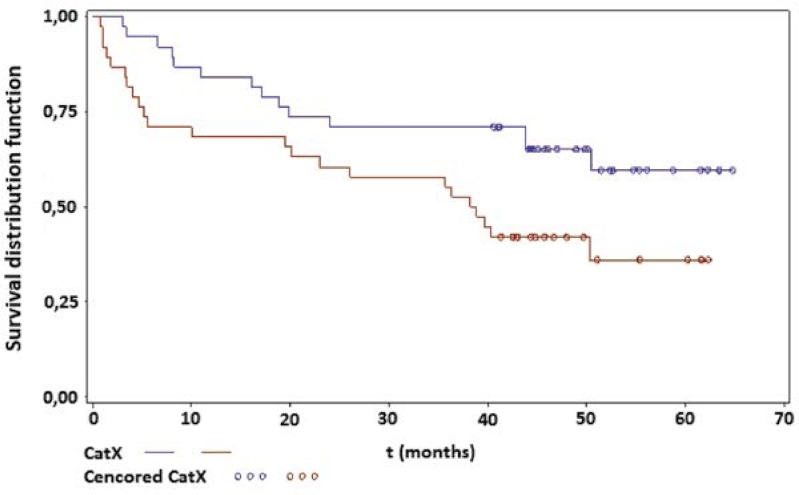
Survival analysis of cathepsin X in serum from patients with CRC. The Kaplan-Meier curve dichotomizing by the median shows the association between total Cat X serum concentrations and patient overall survival. Red colour represents a group of patients with high Cat X levels (≥ to median), blue colour represents a group of patients with low Cat X levels (< to median).

The survival analysis (Figure 1) shows an association between total Cat X serum concentrations and overall survival of CRC patients. The log rank statistics show that patients with higher serum levels of total Cat X have shorter survival than the patients with lower total Cat X serum levels (p=0.028; HR=2.08 (95% CI: 1.07–4.05)). On the continuous scale the difference was not significant (p=0.19; HR=1.48 (95% CI: 0.83–2.64)).

In immune cells Cat X localizes in lysosomes predominantly as a pro-enzyme. After cell activation, Cat X containing vesicles translocate towards the plasma membrane and Cat X can be secreted into the extracellular space.^32^ The similar behaviour was demonstrated also in tumour cells.^5^,^18^ During this process, pro-Cat X can be activated by the other cysteine protease cathepsin L.^33^ Enhanced secretion of pro-Cat X rather than the active form seems to be typical for malignant processes, and therefore, the level of total serum Cat X may better reflect the tumour status than the level of the active Cat X alone.^13^,^22^

The question whether tumour cells or activated immune cells contribute to increased extracellular levels of Cat X remains open. The same holds for the role of Cat X in malignant progression, which cannot be the degradation of extracellular matrix, an event, typical for some other cysteine cathepsins since Cat X acts solely as carboxypeptidase and not as endopeptidase. Based on the results on single and double knock-out mice, Cat X was suggested to compensate the malignant potential of cathepsin B^20^,^34^ enhancing cell adhesion, changing the migration mode^6^ or inducing epithelial-mesenchymal transition in tumour cells.^24^ Its increased serum levels may reflect more aggressive tumour cell phenotype which is related to higher risk for relapse and shorter survival of cancer patients.

Our results show that higher levels of total Cat X in serum from CRC patients significantly correlate to shorter overall survival, suggesting the involvement of total Cat X in the late stages of the malignant process. This is in contrast to the results of Sevenich *et al*.^20^, which show that in breast cancer transgenic mice model Cat X was associated primarily with the initial stages of the malignant process and less with the tumour progression and metastasis. The authors showed that Cat X deficient mice had a prolonged tumour-free period compared to Cat X-expressing mice, however, only a trend toward reduced metastatic burden was observed. Only a combined loss of cathepsin B and Cat X led to additive effects including a reduction in the number and size of lung metastases. On the other hand, the study by Hidaka *et al.* showed that amplification of the region 20q13.2 correlated with the metastatic potential and tumour progression of CRC^16^, suggesting the involvement of Cat X in the later stages of cancer. The latter was proposed also by Wang *et al.*, demonstrating that an upregulation of Cat X expression strongly correlated with advanced clinical stage, shorter overall survival and induced metastatic potential of hepatocellular carcinoma.^24^ Moreover, a recent study by Lines *et al.* showed that in pancreatic ductal adenocarcinoma, Cat X expression is downregulated by over-expressed S100P-binding protein, which results in decreased cell adhesion, correlating with lower metastatic potential of pancreatic cancer cells.^35^

Several molecular targets have been proposed either for pro-Cat X or active Cat X which may contribute to progression of cancer. Pro-Cat X binds integrin receptors by RGD motif^36^,^37^ and interacts with heparane sulphate proteoglycans^8^ modulating cell adhesion and motility. Active Cat X was shown to cleave C-terminal amino acids of chemokine CXCL-12^11^ and beta-2 chain of integrin receptors^6^ regulating chemotaxis and a wide range of beta-2 integrin functions, including cell adhesion, proliferation and migration. Active Cat X also cleaves the C-terminal amino acids of gamma-enolase (neuronal specific enolase - NSE)^10^, a tumour associated protein, used as a marker for prognosis and response to therapy in lung cancer and neuroblastoma. As shown in our recent studies^38^,^39^, un-cleaved NSE regulates proliferation and survival of neuronal cells, whereas the presence of active Cat X significantly impairs the tropic function of gamma-enolase. However, the same effect has not been demonstrated in tumour cells. We may hypothesize that pro-Cat X and active Cat X have the opposite role in malignant progression and the ratio between the pro-Cat X and active Cat X could direct tumour promotive or suppressive action.

To summarize, total serum Cat X could be a useful prognostic indicator to determine overall survival of patients with CRC, serving to optimize therapy and patient care. However, our study based on 77 CRC patients is a preliminary one and a larger group of patients needs to be analysed in a multivariable model to confirm our results.

## Figures and Tables

**TABLE 1:** Subject characteristics

**CRC patients**	**Subjects, n (%)**

Gender	
Female	37 (48.0)
Male	40 (52.0)
Age Group	
40+	3 (4)
50+	10 (13)
60+	17 (22)
70+	26 (34)
80+	21 (27)
Localisation	
Right colon	23 (29.9)
Left colon	34 (44.2)
Rectum	20 (26.0)
Cancer Stage	
I	9 (12.5)
II	32 (44.4)
III	16 (22.2)
IV	15 (20.8)
not specified	5 (6.5)

**Adenomas**	

tubular	45 (58.4)
tubulovillous	16 (20.8)
villous	1 (1.3)
serrat	1 (1.3)
not specified	14 (18.2)
Size	
< 1 cm	41 (53)
> 1 cm	33 (42.8)
not specified	3 (3.8)

**Non-malignant finding**	

Diverticulosis	72 (93.5)
Haemorrhoids	4 (5.2)
Inflammatory bowel disease	1 (1.3)

**Healthy persons**	

	77 (100)

**TABLE 2:** Descriptive statistics for total Cat X concentrations. Values of total Cat X in sera of the colorectal cancer, adenoma, non-malignant findings and the control group of healthy persons.

**group**	**N**	**median (ng/mL)**	**minimum (ng/mL)**	**maximum (ng/mL)**	**mean (ng/mL)**	**SD (ng/mL)**
colorectal carcinoma	77	16.3	5.9	40.4	17.4	7.0
adenoma	77	17.1	4.1	40.0	17.8	6.5
non-malignant findings	77	17.4	2.0	61.5	18.5	8.7
healthy persons	77	17.1	5.4	99.5	18.8	11.4
